# The effects of folk-dance in schools on physical and mental health for at-risk adolescents: a pilot intervention study

**DOI:** 10.3389/fspor.2024.1434661

**Published:** 2024-09-04

**Authors:** Elizabeth Jochum, Ditte Egholm, Anderson Souza Oliveira, Stine Lindahl Jacobsen

**Affiliations:** ^1^ Department of Communication and Psychology, Aalborg University, Aalborg, Denmark; ^2^ Department of Materials and Production, Aalborg University, Aalborg, Denmark

**Keywords:** folk-dance, at-risk adolescents, mental health, physical health, well-being, physical activity, arts & health

## Abstract

We present the findings from a pilot study to evaluate the effects of a six-week adapted folk-dance intervention on physical and mental health for at-risk adolescents conducted in schools. At-risk adolescents are at particular risk for sedentary behavior, poor mental health, and lower quality of life, and are likely to benefit from motivating and health-promoting activities such as dance. However, it can be challenging to conduct and evaluate evidence-based interventions with this population. We conducted a convergent parallel mixed-method design using pre-post measures of mental well-being, as well as pre-post measures using inertial measurement units to assess physical activity during a 6-week adapted folk-dance intervention. At the completion of the study, we conducted semi-structured interviews with all stakeholder groups. We observed significant improvements in mental well-being, as indicated by increased WEMWBS and MHC-SF scores, while the UCLA score showed no significant change, with these outcomes independent of age and gender. Furthermore, at-risk adolescents reduced the time spent in stationary/resting position, while their heart rates were also reduced by ∼15% in such conditions. Our results suggest that at-risk youth who participated in adapted folk-dance became more enthusiastic and showed more willingness to move over the course of the intervention. Quantitative results were supported by interviews, which found that participants responded positively to the adapted folk-dance classes, and reported both elevated physical exertion and high levels of enjoyment. The mixed-method research design also provided insights into the suitability of data collection methods for this hard-to-reach population. We report on these outcomes, including best practices for working within schools on health-promoting physical activities.

## 1 Introduction

Physical inactivity is a major global health challenge, particularly among adolescents. A report by the WHO found that 81% of adolescents worldwide are insufficiently physically active (1), and do not experience the enjoyment and social, physical, and mental health benefits of regular physical activity (2). The association between physical activity, sedentary behavior, and mental health for adolescents is well established (3–5). Higher levels of physical activity are associated with better health-related quality of life, while sedentary behavior is linked to lower health-related quality of life among adolescents (6). Poor physical health in adolescents is often associated with poor mental health, as adolescents that exhibit sedentary behaviour will also experience higher levels of anxiety and depression, as well as lower self-esteem, self-image, and life satisfaction (5, 7). These patterns often continue into adulthood: mental health outcomes of participants with predominantly internalizing problems in childhood or adolescence are often poorer, report lower life satisfaction, decreased psychological health, and physical health-related quality of life during young adulthood (8). The lack of physical activity associated with poor dietary habits places adolescents at greater risk of co-morbidities such as obesity, cardiovascular diseases, type II diabetes, chronic kidney diseases, various types of cancer and musculoskeletal disorders (9). Furthermore, sedentary behaviour is associated with poor cardiorespiratory capacity, leading to increased resting heart rate and greater stress to the cardiovascular system during more vigorous physical activities (10). Ultimately, performing simple walking might become difficult for obese adolescents (11), possibly leading to a negative cycle of physical inactivity. In short, poor physical and mental health during adolescence has been shown to contribute to an increased incidence of obesity, depression, anxiety, and poorer quality of life throughout adulthood (3). Therefore, it is imperative that society focus on initiatives to increase physical and social activities, for both healthy and at-risk adolescents for whom there is relatively little good-quality data available (5, 8, 12, 13).

Schools offer an ideal arena for health-promoting physical activities, particularly for at-risk or vulnerable youth. At-risk adolescents may be defined as those who due to professional, personal, or social reasons are educationally and socially-demographically disadvantaged, and/or those have already had contact with hospital system or social services due to mental health or behavioral disorders, or due to other circumstances have been designated vulnerable (14). Intervention research that aims to combat sedentary behavior, increase physical activity, and promote mental health for adolescents typically focus on exercise and sports (15–19). However, dance has also been shown to promote physical activity and social interaction. Studies show that structured dance activities can significantly improve physical and psychological health (20–23), as well as the potential to counteract loneliness, social isolation, and sedentary behavior across populations, including developing social and emotional skills (24) and reducing depressive symptoms (25). In their systematic review and meta-analysis of dance and structured exercise interventions, Fong et al. found that structured dance activities of any genre are equally and on occasion more effective than other types of structured exercise for improving a range of health outcome measures (26). However, high-quality research in the area is still scarce, with significant challenges such as low statistical power, insufficient sample sizes, lack of follow up measures, and other research biases prevalent throughout the literature (23). Moreover, most dance studies are frequently conducted with older adults (27–31), whereas far fewer studies focus on at-risk adolescents, a population that is at particular risk for sedentary behavior and social isolation (5). Of the few studies focused on adolescents, the majority focus on female adolescents. Staiano et al. reported that dance-based exercise activities for obese adolescent females reduced body fat and mineral density, as well as improved motor coordination, agility, aerobic and anaerobic capacity, strength, and flexibility (32). Folk-dance and social dances were found to promote greater loss in body fat when compared to a classical physical exercise program (33), while Duberg et al. found that dance was an enjoyable and empowering activity that fostered emotional expression, supportive environments, and self-acceptance for females with internalizing problems (21).

Dance is a multimodal activity that combines physical and cognitive activity through physical activity, musical listening, and social interaction (34). However, the interplay between physical and psychosocial research outcomes of dance interventions appears to be under addressed in the literature. Within dance and health research, there are several categories of dance interventions, including diverse genres of dance (e.g., ballet, jazz, folk, West African, hip hop); exercise dance; and Dance and Movement Therapy (DMT) [also known as Dance Movement Psychotherapy, or Movement Psychotherapy (MP) in the UK], which is a form of psychotherapy that leverages the creative uses of movement and dance, and led by credentialed professionals and conducted either in group or individual sessions (35). Most of the clinical research concerning dance and mental health has been conducted with DMT. Studies show that DMT can reduce internalized symptoms such as anxiety and depression among adolescent girls, reducing the concentration of dopamine and increase serotonin levels (22) and improving the mood states of adolescents suffering from a variety of psychiatric illnesses (36). DMT interventions have also been shown to support and improve academic performance (37), as a promising treatment for neurological disorders (38), to manage and prevent stress in children and adolescents (39), and to support quality of life and manage depression and anxiety for diverse patient populations (23, 40), including managing stress and anxiety for cancer patients (41). A DMT study by Punkanen et al. with working age people with mild, moderate, or severe depressive episodes found that following 20 DMT group sessions improved depression, lowered anxiety, decreased the measure of neuroticism, and increased measures of extraversion and life satisfaction. These are promising results, but the authors note their study was marked by similar methodological challenges facing other DMT studies –lack of a control group, low sample size, and no follow up measures. While this study evidences the strong link between movement/motion and emotions, there are still important knowledge gaps such as the links between dance, mental health, and physical health, especially for hard-to-reach populations like at-risk youth.

Furthermore, the prior published outcomes on the impact of dance and DMT on health-related outcomes have considerable limitations, the clinical methods are not always clearly described or reproducible, and the findings are of limited generalizability. For example, one of the larger published studies on DMT and mood conducted by Andersen et al. (36) retroactively examined the effects of a single DMT on mood changes for adolescents within a larger psychotherapeutic day treatment program conducted at a hospital. But the study is a retrospective analysis based on a chart-review study, and mood changes were assessed using a visual guide with an emotion picture and adjectives. Furthermore, no details of the actual intervention are described in the study. A study by Salihu et al. with internally displaced persons with depressive symptoms found that DMT sessions significantly reduced depressive symptoms from severe to mild, as well as reduced anxiety and stress, with high recruitment and relatively low dropout rates (25). However, the authors caution that the treatment effect might be overstated given the imbalance in the baseline characteristics of the groups: the baseline scores showed higher depressive symptoms in the intervention group compared to the control group. Limitations like this are not uncommon in dance and mental health research. Koch et al.'s systematic review on the health-related psychological effects, including clinical outcomes of dance and DMT studies, document the promising effects as well as the significant methodological shortcomings in the published research (23).

Conducting intervention and prevention research for at-risk adolescents is widely known to pose specific challenges when it comes to recruitment, participation, and engagement (8, 42, 43). Efforts to improve adolescent physical activity surveillance, research, intervention implementation, and policy development are urgently needed (13), as well as change mechanism research on those factors that support increased physical activities for at-risk youth (12). More good quality, evidence-based research is needed to fully understand the impact of dance on the combined physical and mental health and well-being for at-risk adolescents. A deeper understanding of this relationship can help inform more engaging and motivating dance programs, and a better understanding of the underlying mechanisms of change. Interpreting physical or psychosocial variables is highly complex even in isolation, and combining the two requires careful design of study protocols and multi-factorial approaches to understand and explain research outcomes. Therefore, the aim of this pilot study was to evaluate the effects of a six-week adapted folk-dance program on psychosocial (e.g., loneliness, mental health, well-being) and physical fitness (e.g., physical activity levels, heart rate) outcome measures in at-risk adolescents. We created a mixed method approach to assess holistically the progress of the adolescents, while taking into account interactions with school staff, dance instructors and musicians to adapt to the needs of the participants. Our main hypothesis was that adapted folk-dance with live music would improve mental health and well-being for at-risk adolescents, and would also improve physical fitness by increasing the amount of movement performed and reducing heart rate at low-level physical efforts.

## 2 Methods

### 2.1 Design

Pilot studies are useful for testing the effectiveness of proposed interventions and evaluation instruments and can help to identify potential issues before committing to a full-length research study. They can also be useful for gaining insight into populations that are underrepresented in the literature or difficult to study (44). We conducted a mixed-methods, parallel convergent research design using pre-post study measures within a post-positivistic paradigm. The intervention consisted of six dance sessions (one session per week, 90 min total with a half hour break) during school time. Participants answered a questionnaire before and after each session to provide data related to mental health, wellbeing, and loneliness. In sessions two and six, participants wore an inertial measurement unit (IMU) to measure their physical activity level and heart rate. Moreover, participants, teachers, and folk-dance professionals were interviewed after the conclusion of the intervention, to explore the experience of participating from multiple perspectives.

### 2.2 Recruitment

The intervention took place at alternative schools that cater to a broad target group that are high risk for poor mental health, such as young people who have been in contact with the hospital system due to a mental disorder, have substance abuse problems, cognitive impairments, behavioral disorders, limited schooling, dysfunctional families, or otherwise require professional assistance (14). Participants were recruited during informational sessions held during school hours at three different schools offering professional development for young people who have been deemed unable to follow standard public schooling or enter employment due to professional, personal, or social reasons (14).

During the information sessions, participants were introduced to the research design and data collection methods, including the IMU handling and placement and questionnaire formats. Participants were eligible to participate if they were currently enrolled in the school, aged 16–25, and spoke fluent Danish. The exclusion criteria for the movement analysis/heart rate study included under the age of 18, inability to stand and/or walk independently, lack of ability to cooperate, and participation in medical trials or other training interventional trials throughout the study period. The project was conducted in accordance with regional guidelines for medical ethics research and was approved by the regional medical ethics committee (VEK, Journal numbers: 2022-000764; 20230001). Written informed consent was obtained prior to participation.

### 2.3 Participants

A total of 63 adolescents (35 females aged 16–28 years/28 males aged 16–26 years) agreed to voluntarily participate in the adapted folk-dance activity, of whom a total of 46 students elected to participated in the research study. Of these 46, only 16 completed both pre- and post questionnaires, and only 6 of those wore activity sensors. Additionally, 6 staff from the schools and 6 dance/music instructors were interviewed, but they did not participate in the questionnaires. Several adolescents have diverse diagnoses, including mild to severe levels of stress and anxiety-related challenges, among other mental health and physical problems. One student was deaf and attended classes with an interpreter. Thirty-five at risk adolescents answered the questionnaires on mental health and loneliness before the intervention, and twenty-six answered the questionnaires after the activity. Only 16 at risk adolescents (∼25% of the study sample) answered the questionnaires both before and after the intervention. Pre/post mental health questionnaire results are based on these 16 participants. Six at risk adolescents (∼10% of the study sample) volunteered to wear the IMU sensor, and a total of 35 at risk adolescents (∼52% of the study sample) agreed to be interviewed after the intervention. Based on the interview feedback from 35 participants, mean participation in the six sessions was 70% (Table 1). Furthermore, six staff members and six artists (2 dance instructors and 4 musicians) agreed to be interviewed. Complete demographic information in the data collection in Appendix 1.

**Table 1:** Study participants.

Questionnaires (both pre/post)	Interviews	Activity sensors
46 at risk adolescents (16)	35 at risk adolescents 6 staff 6 artists	6 at risk adolescents

### 2.4 Folk-dance intervention

Each weekly session consisted of a 90 min adapted folk-dance workshop led by professional folk-dance instructors and musicians with experience working with vulnerable populations. The intervention was offered as an integrated part of daily activities and occurred during regular school hours. The dance instruction and live music were adapted to the needs and capacity of the participants. Each session consisted of three parts: two 30-minute dance sessions and one 30-minute break in between. During the dance sessions, one instructor welcomed the group and provided instructions on specific folk-dances, adapting to the participants’ needs and energy levels along the way. A team of three musicians accompanied with live music that was also adapted, for example, slowing down or speeding up, depending on the capacity of the participants. Young people, supporting professionals, and teachers danced together following the folk-dance instruction and music. Refreshments were served during the break, during which there were storytelling and group singing activities.

The movement of the folk-dance classes consisted of ever-changing dynamic formations and included a combination of group dancing, partner dancing, and dancing in small groups. The movement was always centered on moving together as an individual or with a partner and was only on a few occasions in smaller groups of four pairs. Group dancing usually took place in a circle or else in parallel lines standing across from each other. The dance classes would often build in intensity with starting slower and building in pace and difficulty. The focus was rarely on mastering the technical elements of folk dancing and more on the feeling of togetherness, mastering the pace, directions and partnering. The dance classes progressed during the six sessions, with some of the choreographies evolving by introducing variation or more complex movement patterns (e.g., the different steps or directions added). The progression involved altering the pace (faster or slower) or the possibility to choose one's own variation with your partner such as faster turns, covering a greater distance or modifying the position of the hands (called “holds”). While some dances were repeated across all 6 sessions, new dances or elements (such as walking in long lines covering more space than when moving in circles) and small variations were added each session. The dance is considered adapted as the musicians and dance instructors received training in adapting the dance instruction and music to the needs of the study population. Specifically, a separate intervention study was run in parallel by the dance instructors and musicians working in assisted living facilities for adults and elderly with cognitive impairments. Although the music and dance followed the same overall structure, the specific accommodations and instruction were tailored to the individuals and groups, and adapted with consideration of their physical and psychological needs and constraints (e.g., offering alternatives for people who do not want to or are unable to hold hands).

### 2.5 Outcome measurements

#### 2.5.1 Mental health, loneliness and well being

Mental health was assessed using three different questionnaires to provide a more comprehensive and nuanced picture of the participants’ mental health (45). The first is the Mental Health Continuum Short Form (MHC-SF), which measures emotional and functional (social) dimensions, as well as psychological well-being. The assessment is based on the presence of one or more symptoms measured by the three dimensions, leading to a classification of complete or incomplete mental health determined by the number of symptoms. Mental health is termed “flourishing” for complete mental health and “languishing” for incomplete mental health, with moderate mental health in between (46). MHC-SF was translated into Danish by the Danish National Institute of Public Health and verified (47). The second questionnaire assessed loneliness through the UCLA Loneliness Scale (Version 3) (48). The 20-item scale examines objective and subjective measures of loneliness as well as feelings of social isolation. The scoring is based on a four-point scale, with higher scores indicating a greater expression of loneliness. The scale has shown high internal consistency and test-retest reliability with a coefficient alpha of over 0.90. A Danish translation has been validated previously (49). The third questionnaire assessed mental health through the Warwick-Edinburgh Mental Well-being scale (WEMWBS). The scale is designed to capture a broad conception of well-being, including affective-emotional aspects, cognitive-evaluative dimensions, and psychological functioning. The scoring is based on answering seven items on a five-point Likert scale ranging from 1 (no time) to 5 (all the time). The Danish translation of the WEMWBS scale has been validated in Denmark (50).

#### 2.5.2 Physical health

Physical activity levels and heart rate were assessed during the 60 min of dancing for multiple study participants (*N* = 6 simultaneously using inertial measurement units (IMU) with the capability of measuring tri-dimensional linear acceleration and angular rates, as well as electrocardiographic (ECG) signals (Shimmer3, Shimmer Research, Dublin, Ireland). For males, the IMU was fixed using a compatible chest strap provided by the IMU manufacturer. For females, the IMU was fixed on top of the upper region of the sternum bone, firmly secured using medical tape. The IMU weights 0.011 Kg and measured tri-axial accelerations and five electrocardiography (ECG) signals using standard Ag/AgCl electrodes (Kendall H124SG, Cardinal Health, Dublin OH, USA). Therefore, we expected that participants would not be influenced by the presence of the IMU on their bodies during the folk-dance sessions. The ECG electrodes were placed on the upper chest and lower trunk bilaterally, with a fifth sensor being placed on the right low ribcage. Both accelerations and ECG signals were sampled at 512 Hz. Data was recorded continuously throughout the whole 90 min during sessions two and six.

#### 2.5.3 IMU data processing

Firstly, the data from different accelerometers were synchronized through the individual timestamps. The tri-axial accelerations time series were low-pass filtered (3rd Order Butterworth) with a 10 Hz Cut-off frequency (51) and converted from m/s^2^ into *g* forces. Subsequently, the resultant acceleration (ACC) was calculated:

$$\mathrm{ACC}=\;\surd (x^{2}+y^{2}+z^{2})$$

Where, *x*, *y* and *z* are the anterior-posterior, medial-lateral and vertical accelerations (respectively).

Physical activity levels found by computing the mean activity deviation (MAD) from the resultant acceleration as described elsewhere (45). The MAD computed the distance of individual datapoints with respect to the mean of all points in the time window. In this study, the MAD was computed at every 10 s and classified as “rest”, “slow walk”, “normal walking”, “brisk walking” or “jog/running”. The total time in each class was normalized by the total time of the folk-dance activity (% total). In addition, we estimated the total amount of steps performed during the folk-dance activities, by computing the number of acceleration peaks above the standard deviation of the 10-second windows.

The ECG signals were converted into instantaneous heart rate using the manufacturer data acquisition software (Shimmer3, Consensys ECG, Shimmer, Dublin, Ireland). The raw heart rate data was low-pass filtered using a 30 s moving average with steps of 1 s). Subsequently, the heart rate was normalized by the maximum expected heart rate based on the participant's ages, using a widely used formula from the literature (maximum heart rate = 208−0.7*age) (52). Heart rate data were averaged at every 10 s to match the duration of the windows from activity levels. Finally, each 10 s heart rate window was reduced to a single average point. Therefore, every 10 s of folk-dance performance was represented as an activity level and a heart rate value. The heart rate recorded during the “rest” class from the MAD classification is an estimate of the lowest heart rate during the session. However, it is not representative of the possible resting heart rate of the participants, since they were not seated/resting for an extended period of time during these recordings.

#### 2.5.4 Interviews

A total of six focus group interviews were conducted with four to six participants per group (participants *n* = 33). Additional interviews were conducted with teachers (*n* = 4), and folk-dance professionals responsible for the intervention (*n* = 4). All six interviews with participants were held in-person on site at the school. Interviews with teachers, musicians, and dance instructors were conducted online. Participants reported different experiences based on the number of times they participated in the dance sessions, and whether they participated physically in the dance (active participation), by observing (passive participation), or a combination of the two (see Appendix 1). The interview guide was based on a semi-structured interview, in which the interviewer followed a list of topics to be covered but was permitted to pursue lines of discussion that wandered from the guide when necessary (35).

The interviews lasted between 28 and 48 min (average: 36 min); audio recordings were made and transcribed using an automatic transcription tool and following manual corrections and were manually reviewed by the interviewer and corrected for typographical mistakes or spelling errors. Transcripts from the interviews were analyzed independently by three members of the research team: the interviewer who took part in all dance sessions, and two researchers familiar with the study but not present at the interviews. Thematic analysis was conducted of the textual data separately, following the six general steps outlined by Braun and Clarke (53) using a combination of inductive and deductive approaches, and following their 15-point criteria for ensuring good research practice. After the researchers completed this task independently, they met to discuss commonalities and differences, and then repeated the steps together. Textual data was coded, and themes and sub-themes emerged, which were then presented in plenum to the entire research team. Qualitative data analysis was conducted in Danish, and the themes and data were subsequently translated into English by the research team.

### 2.6 Statistical analysis

We investigated the changes observed from pre-measurements to post-measurements across six dance sessions. This analysis employed Analysis of Covariance (ANCOVA), with pre-measurements serving as covariates for WEMWBS, MHC-SF, and UCLA as there were only two measurement time points. The analyses adhered to the principles of intention-to-treat, wherein all relevant available data were included. Statistical analysis was only conducted for the 16 individuals who completed both pre and post questionnaires. Due to the limited size of the participant cohort in this pilot study, adjustments for multiple comparisons were not applied, and the significance threshold remained at 0.05. Regarding step count, MAD and heart rate, only descriptive statistics are presented due to the small sample size.

## 3 Results

### 3.1 Questionnaires

The questionnaire data included eight females with the average age of 19.25 years and with a standard deviation of 1.58. Eight males participated with the mean age of 18.38 years, showing a standard deviation of 0.92. An analysis of demographic variables was examined showing age and gender evenly distributed among participants. We observed a significant increase in the WEMWBS score from pre-intervention (M = 41.88, SD = 13.04) to post-intervention (M = 46.44, SD = 9.73), t(16) = 0.00, *p* < 0.05, with a moderate effect size (*η* = 0.60). There was also a significant increase in the MHC-SF score from pre-intervention (M = 34.63, SD = 16.82) to post-intervention (M = 40.19, SD = 13.55), t(16) = 0.00, *p* < 0.05, with a moderate effect size (*η* = 0.56). The UCLA score showed no significant changes (pre M = 34.06, SD = 6.70; post M = 33.94, SD = 5.07), t(16) = 0.70, *p* > 0.05, *η* = 0.01 (See Table 2). Overall, there was a significant improvement in mental well-being for the at-risk adolescents over time. Loneliness decreased from pre- to post- measure, but not significantly. The ANCOVA analyses did not depend on or covary with either age or gender.

**Table 2:** Mental health survey data.

*N* = 16	Pre-intervention Mean ± SD	Post-intervention Mean ± SD	Effect size (*η*)
WEMWBS	41.88 ± 13.04	46.44 ± 9.74**	0.60
MHC-SF	35.63 ± 16.82	40.19 ± 13.55**	0.56
UCLA—loneliness scale	34.06 ± 6.70	33.94 ± 5.07	0.01

* *p *< 0.05.

** *p *< 0.01.

### 3.2 Resting duration and heart rate

**Figure 1:**
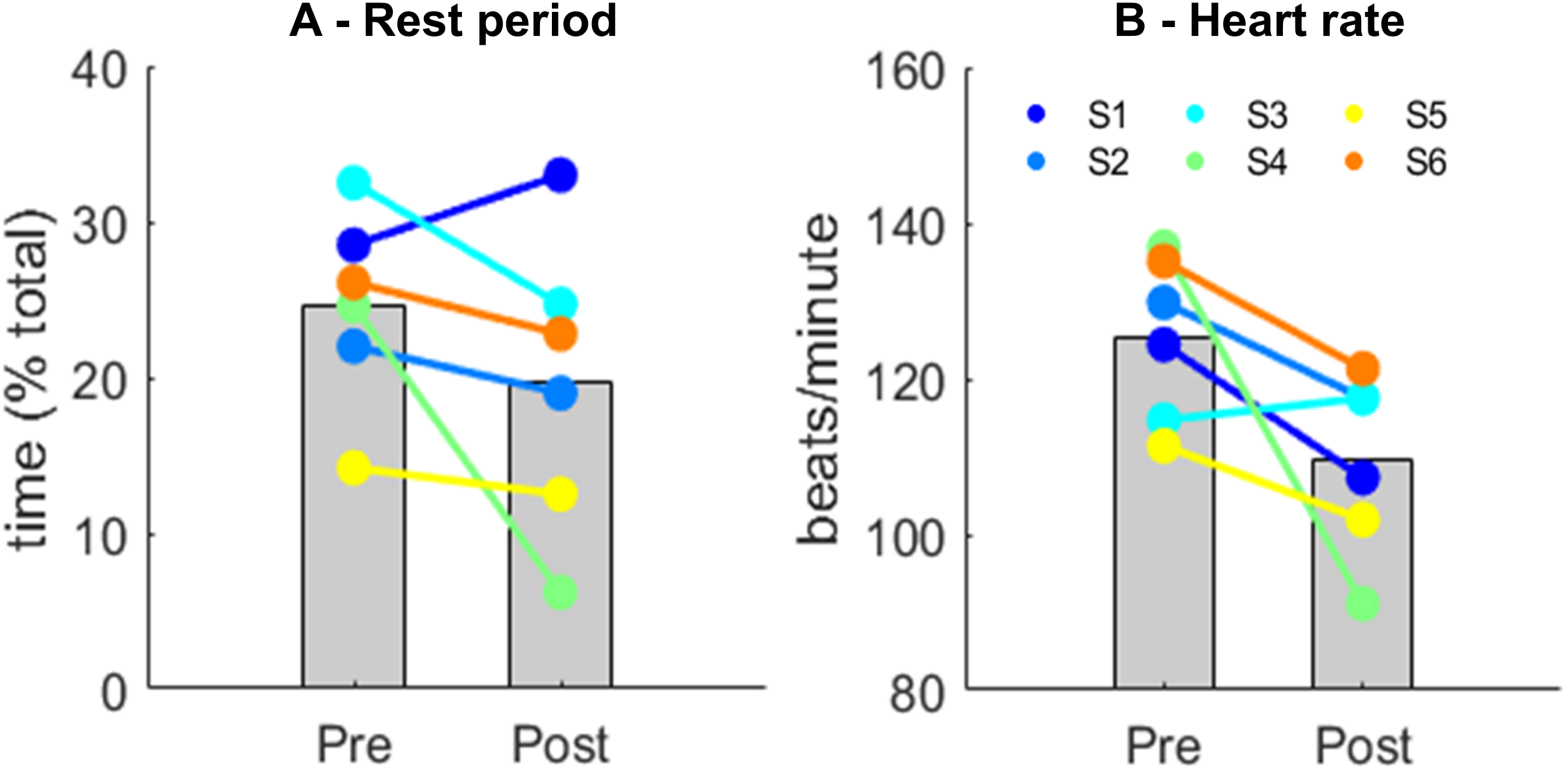
Mean (*gray bars*) and individual data for the duration of the stationary periods **(A)** and the respective heart rate during stationary periods **(B)** before (Pre) and after intervention (Post). Each color represents a participant (S) and their trend from Pre- to Post-intervention.

Data from heart rate and physical activity levels were assessed in the post-test from only four participants, since we had two drop-outs in the post-test. Heart rate data was only collected during the times the participants were actively dancing (approximately 60 min of the 90 min sessions), and no recordings were taken during the singing break. The average active time during the folk-dance sessions was 50 min (range: 44–55 min) across the pre- and post-intervention sessions. The average difference between the total amount of steps taken was ∼5% when comparing pre- (2,987 ± 434 steps) and post-intervention (2,773 ± 138 steps). The stationary periods throughout the sessions (Figure 1A) and its respective heart rate (Figure 1B) were reduced by ∼25% and ∼15% from pre- to post-intervention, respectively.

### 3.3 Activity levels and heart rate

**Figure 2:**
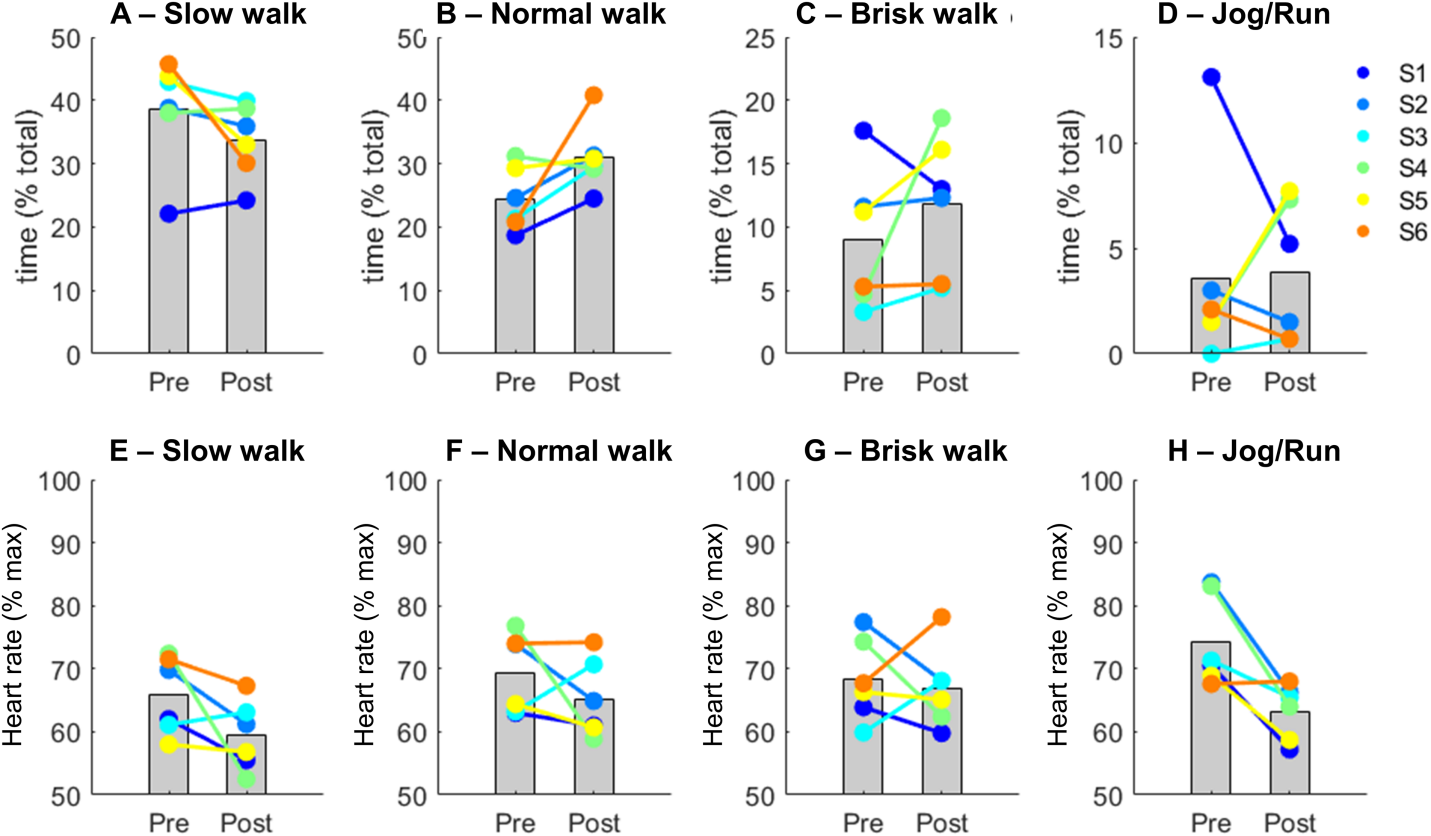
Mean (*gray bars*) and individual data for the duration of physical activity levels (*upper row* **(A–D)**) and their respective heart rates (*bottom row* **(E–H)**) before (Pre) and after intervention (Post). Each color represents a participant (S) and their trend from Pre- to Post-intervention.

Overall, the heart rate of participants ranged between 60% and 70% max. There were reductions on the time participants spent at a slow walk activity level (−15%, Figure 2A), whereas the time at normal walk (Figure 2B) and brisk walk (Figure 2C) increased by 22% and 24% respectively. At the Jog/Run activity level there were highly inconsistent patterns across participants, which on average increased their time by 8% (Figure 2D). Regarding heart rate, there was an overall reduction across all types of activities (slow walk: −11%; normal walk: −6%; brisk walk: −2% and Jog/Run: −17%; Figures 2E–H). However, there were inconsistent patterns across participants, especially for the brisk walk (Figure 2G).

### 3.4 Interviews

Of the 63 study participants in the study, 35 took part in post intervention interviews along with dance instructors (*n* = 2), musicians (*n* = 4), teachers/staff (*n* = 6) at the schools. Five main themes were identified: (1) Relation, (2) Progression, (3) Physical Experience, (4) Facilitation, (5) Motivation & Participation. Table 3 presents the main themes and subthemes identified through thematic analysis.

**Table 3:** Interview data.

Theme 1: Relation	Theme 2: Progression	Theme 3: Physical experience	Theme 4: Facilitation	Theme 5: Motivation & participation
Openness	Motor skills	Physically challenging	Adjusting & flow	Expectations
Equality	Confidence	Touch anxiety	Roles	Self improvement
Self-consciousness	Social mastering	Live music & dancing	Inclusivity & flexibility	Chaotic and unfamiliar
Community	Endurance		Supportive & non-judgemental	Mood

#### 3.4.1 Relation

Within this main theme, four subthemes were identified: openness, equality, self-consciousness, and community. Initially, one of the main challenges for participants was interacting and being physically close with other participants whom they did not know. Such experiences were perceived as potentially anxiety-inducing and transgressive because they require participants to engage in unfamiliar activities with new people, to learn new and unfamiliar choreography together with strangers, and to physically interact with each other (e.g., holding hands, partner dance). Participants reported that these initial stressful feelings changed relatively quickly over time, and they promptly grew accustomed to switching dance partners and did not find the experience frightening:

*Maybe it*’*s because I can find it difficult to be close to someone that I don't know very well. It could be challenging sometimes if you changed partners and ended up with someone you had never talked to before. Then there is dancing with them, it could seem just a little uncomfortable. But then it was a safe space anyway. So it wasn't exactly that I thought “now I'm going to leave.” But it could be a bit challenging sometimes. But I managed it very well*. -Participant

Participants also commented that it was enjoyable to have the teachers participating in the dance sessions, because it provided a new way to relate to them on equal footing:

*Your relationship with the teachers didn't get any worse anyway. You could almost have a bit of fun afterwards when you had danced with them. It was kind of funny that you had been in the same boat with them. But you were on the same level. They weren't better at anything than you were yourself.* -Participant

Both participants and teachers commented on the sense of being part of a community and of having fun failing together:

*It*’*s always the social thing. It is always the social aspect that is the focus.* -Teacher

*You are not exposed or made fun of, or anything of the sort. We laugh together.* -Teacher

*There is also a huge sense of community. It's not like you dance on your own, you dance with each other… Already after the first time, you felt a bit that we were in the same boat. The thing about folk dancing is that it brings about such a sense of community. I don't know why. But it feels like a fun dance. You laugh a lot*. -Participant

#### 3.4.2 Progression

Within this main theme, four subthemes were identified: motor skills, confidence, social mastering, and endurance. The subthemes reflect the variety of ways that study participants participated, and whether or not they experienced a change in their progression. While many participants self-reported an experience of progress and improvement, others did not:

*Yeah, so for me, it got easier to dance with some new people… it wasn't something I was as afraid of. So for me, there was a progression*. -Participant

*When it comes to learning a new dance, I feel like I have to do it several times to get the hang of it. I can quickly become nervous about whether I'm doing it right. It was actually a bit difficult to keep up. But on the other hand, after a while, it was like—okay.* -Participant

*Yeah, well, it was still uncomfortable from time to time. But after the first time, maybe you were also a bit prepared to know that you had to dance with a person you probably didn't know. So, it was maybe not as bad the second time as it was the first time*. -Participant

The teachers’ and folk-dance instructors’ observations were useful in identifying subtle changes in participation that may have not been captured in self-reporting by the participants. For example, one dance instructor stated:

*It was one of the last sessions, where there had been a young man who had just sat—just sat and not participated at all. But during the singing portion, I noticed so clearly that his foot just followed the rhythm. So, somehow, it was clear that he had “been there” after all.* -Dance Instructor

#### 3.4.3 Physical experiences

Within this main theme, three subthemes were identified: physically challenging, anxiety and touch, and live music and dancing. The subthemes show that although many participants found the dance activities physically demanding—participants frequently became out of breath and perspired a lot—they also found themselves to be energized by the dancing. One deaf participant who participated with a sign-language interpreter commented:

*At first, I felt it was actually quite hard. I had to look at the dance instructor and the interpreter. It was actually a bit difficult. I used a lot of resources to concentrate. Even if you had fun and thought it was fun. But afterwards, I was completely tired and done. I thought it was hard physically and mentally. I thought it was cool that I was there. And there were also many who praised me and said that it was great that I was there. Whether I had an interpreter or no interpreter, I still participated. I tried to keep up as best I could*. -Participant

The dance instructors also observed that the dance was physically challenging for some participants:

*What we found was that the young people*’*s fitness—or rather their* lack *of fitness—was simply a challenge. They became really, really tired, really, really fast. … There were many people who simply sweated and gasped after just half an hour of dancing.* -Dance Instructor

Some participants commented directly on the experience of dancing to live music and how it supported the dancing.

*I think it*’*s strange music to start with. But after you begin to learn the dance to those songs, I think it fits quite well together…I liked it. Not because I've ever heard anything like it before. But there was a good rhythm to it, and then you could make a stomp on the ground, and snap and count. I thought it was really good.* -Participant

#### 3.4.4 Facilitation

Within this main theme, four categories were labeled: adjusting and flow, roles, inclusivity and flexibility, and supportive and non-judgmental atmosphere. There was consensus among the teachers and dance instructors that there was an overall supportive atmosphere that was customized to the needs and challenges of the individuals and each school group:

*We should remember not to set too high expectations for (the students). It*’*s not something they have to necessarily be able to do. There are no steps they have to master. So you just adapt it based on who you're dancing with. You can still be part of a community even if you're not actively participating, even peripherally.* -Dance Instructor

Many participants commented on the inclusive and non-judgmental space and how it made them feel safe in the dance session where the learning and progression just happened in a natural flow:

*It was nice that everyone was included* …*I felt like there was room for everyone, including those who might not want to dance. It was just nice like that.* -Participant

*That it was so fun while learning it. It was so impressive that we didn't just stand there*… *It just happened. I liked that.* -Participant

Teachers, dance instructors, and musicians understood that non-verbal guidance and being a role model was important to support the young participants and sustain their motivation.

*I definitely experienced that it was important for us to be there. Because if they were going to do it, then we should too. Yes, I think so. Because then they would also feel more inclined to dance: “Because if she*’*s dancing, then we should too,”* -Teacher

*He [the dance instructor] was very positive and also very good at teaching us like that. And he was also good at noticing if you didn't understand at all what he said. Then he just demonstrated it with you so you felt like you could do the movements at least to somewhat.* -Participant

#### 3.4.5 Motivation & participation

Within this main theme, four subthemes were identified: expectations, self-improvement, chaotic and unfamiliar, and mood. It was clear that many participants felt reluctance towards dancing folk-dance at the beginning, and their initial interest and expectations were quite low. Some participants described how this first impression changed quite quickly, while others continued to experience some discomfort in the unfamiliar setting.

*Just when I heard about it, I thought it wasn't something I should try. I don't know why. It*’*s folk dancing. Many people. It wasn't exactly the coolest thing, I thought when I heard about it. But it was actually a lot of fun. It was exciting.* -Participant

*It was much better than I expected. It was fun, and it brought good vibes… and we laughed at each other, and with each other.* -Participant

Some participants welcomed the challenge and saw it as an opportunity to improve, and some participants expressed how the dancing changed their mood and the mood of others.

*It was a bit boundary-crossing, yeah. But I think they were some good boundaries to cross*. -Participant

The main themes identified in the interviews provide insights into how the intervention was perceived and experienced by participants, teachers, dance instructors and musicians. This makes it possible to consider these results alongside the quantitative data to gain deeper insight into the impact and underlying mechanisms of change.

## 4 Discussion

The primary aim of this study was to pilot an adapted folk-dance intervention in schools for at-risk adolescents as a health promoting activity to strengthen mental health, physical fitness, and well-being. Our main findings suggest that the adapted folk-dance had a positive effect on mental well-being and physical fitness. For those participants who completed the pre-post mental health questionnaires, the results suggest that the adapted folk-dance had a positive effect on their mental well-being. There was a significant increase in both WEMWBS and MHC-SF scores from pre- to post-intervention, indicating an improvement in their mental health status. The moderate effect size (*η*) indicates that the intervention had a notable impact on the participants. The cut-off score for WEMWBS is 42, which adds an additional dimension of significance to the results. Prior to the dance activity, the average WEMWBS scores for young participants were below 42 (M = 41.88, SD = 13.04), indicating a low level of mental well-being. After the intervention, average WEMWBS scores significantly increased above the cut-off level (50), which is encouraging. There were no significant changes in the self-rating of loneliness and social isolation (UCLA Loneliness Scale) following the intervention. For those participants who wore IMU sensors, at-risk adolescents increased by 20%–25% the time performing moderate or high-intensity movements at the end of the intervention. Moreover, there were reductions in average heart rate at both low- (−11%) and high-intensity movements (−17%). The results from the interviews revealed that the dance sessions were experienced as providing a strong sense of community and equality across participants, teachers, dance instructors, and musicians. Most importantly, participants experienced a greater sense of belonging during the sessions. Taken together, our results show that at-risk adolescents were more enthusiastic and willing to move more at the end of the intervention, and they experienced increased well-being and sense of belonging. The increase in activity time in the post-test measurement may be a combination of voluntary effort to maintain the engagement during the dancing activity, as well as due to the improved physical fitness. This means that participants were more motivated to move and were also better prepared (physically) to move. Overall, these results are encouraging and suggest that the intervention could be an effective method for enhancing mental well-being and promoting physical health for this population. Although we are encouraged by our findings, they must be interpreted with caution as there is not significant sample size to to carry out a well-powered statistical analysis.

While device-based research is an important aspect of physical activity monitoring, collecting enough good quality data for this population can be difficult. We found that while many participants across the three schools were interested in participating in the dance sessions, very few of them wanted to be directly involved in the research dimensions of the study that would require them to fill out survey data and wear an IMU. We also found absenteeism to be a major hurdle for collecting good quality data, making it difficult to gather pre/post data for the all the participants who agreed to the study. We strongly agree with van Slujis et al. (43) that research on school-based and community-based interventions would do well to focus on mechanisms of change (mediation), implementation (i.e., adoption, dose delivered, reach, fidelity, and sustainability), and the determinants of implementation (e.g., feasibility, adaptability, and acceptability) in addition to device-based research (43). Such insights are vital if interventions are going to be realistic and sustainable over the long term. Accordingly, we opted for a mixed methods research design that would enable us to both conduct device-based research as well as evaluate change mechanisms with a focus on implementation, thereby gaining deeper insights into the relationship between physical activity and mental health and well-being effects of adapted folk-dance. Our study thus confirmed the difficulties involved with recruiting and collecting sufficient statistical data for this population previously identified in the literature (2, 42, 52). We discuss these challenges in more detail in the Limitations section. Overall, the results and experiences from this pilot study are promising, and also raise important considerations that can help inform future interventions in schools to promote health and well-being, and promote authentic engagement from all stakeholders.

### 4.1 Mental health (well-being, loneliness, and social isolation)

During the interviews, participants self-reported experiences of poor mental health and social anxiety. They also face greater vulnerabilities due to their socially-disadvantaged position. The improvements observed in the scores from two of the three self-reported mental health measures (WEMWBS and MHC-SF) indicate potential for adapted folk-dance in school as a promising intervention to promote physical activity and to strengthen mental health and well-being. We know from research that functioning well in life not only involves individual emotional and psychological well-being, but also refers to how well individuals function in their respective communities and society (46, 54). Research also shows that dance can have a positive influence on mental health by reducing anxiety and depression (55), reducing the concentration of dopamine and increase serotonin levels (22), and managing and prevent stress for children and adolescents (39), and improving psychological well-being for adolescent females (20). Our study focused of the effects of adaptive folk-dance on mental health for at-risk adolescents addresses a gap in the literature for which there is relatively little data available. Although no significant changes were observed for loneliness, this could be explained by the fact that the pre- mean for this group was already relatively low (pre M = 34.06, SD = 6.70), suggesting that their initial level of loneliness was not clinically significant (normally scores of 20–34 denote a low degree of loneliness). The improved well-being could be related to reduction in anxiety levels, as well as the increased sense of belonging during the activities, could explain the reduced the anxiety levels. The mental health and well-being survey data is supported by interview data which found that participants responded positively to the intervention overall, and reported high levels of enjoyment, positive shifts in social participation, and mastery of social skills. The qualitative data from the teachers and dance instructors further supports the survey data, as they observed that study participants showed more willingness and comfort moving in a group. We are therefore encouraged by the positive changes in mental health.

### 4.2 Physical fitness (heart rate, activity levels)

For those participants who wore IMUs, we found that participants reduced the time spent in stationary/resting position, while their heart rates were also reduced by ∼15%. Such results may suggest that the adolescents who participated in adapted folk-dance became more enthusiastic and willing to move more at the end of the intervention. It is well known that sedentary behavior increased the risk of depression (56), whereas physical activities have been proven as effective in reducing sympathetic nervous system and hypothalamic-pituitary-adrenal axis reactivity (16). A corroborating result from our study is that participant's heart rates during the resting/stationary periods was ∼25% lower at the end of the intervention. It has been shown that specific types of exercises such as endurance training and yoga can reduce resting heart rate (57). Although we did not access resting heart rate, our assessment during the resting periods of the folk-dance activity may suggest that resting heart rate can decrease following such type of intervention. Such a result may be a combination of slightly improved aerobic fitness due to the intervention, as well as a reduced anxiety feeling throughout the sessions.

Our study demonstrated that those at-risk adolescents who wore sensors increased time spent in moderate activities, as they increased their time in the normal walk and brisk walk categories (Figures 2B,C). Interestingly, there were also slight reductions in heart rate during these activity classes, demonstrating that adolescents participating in the adapted folk-dance intervention increased their engagement in moderate movements while experiencing lower heart rates. It is plausible that the beneficial effects of the intervention on any possible anxiety issues contributed to a positive modulation of parasympathetic function, such as the augmentation in vagal tone demonstrated in animal studies (16). Improved parasympathetic function can optimize cardiorespiratory function, ultimately reducing heart rates during exercises. Folk-dance has been shown to increase overall physical fitness in older adults (58) and female adolescents (20), and our study demonstrates that it can impact the physical fitness of at-risk adolescents, as they may be able to improve physiological functions along with cognitive/psychological benefits from the group classes.

We observed a notable drop in the amount of resting time and also in heart rate during Session 4. The notes from the observational data show that this session included more technically complex dances, which were observed to be challenging for the participants and required more focus. Therefore, it is plausible that the activity was more focused on mild movements rather than vigorous movements, reducing overall heart rate and increasing overall step count due to the longer periods participants were moving.

Although participants experienced certain physical challenges and fatigue during dance activities, they also reported an increase in confidence and a sense of achievement. This was supported by observations from dance instructors, who noted small but meaningful progress among participants, which may not always be captured by quantitative measurements. Again, this seems to align with the quantitative measurements for mental heath and well-being.

### 4.3 Combined evaluation and experiences—A 360° view

In the subsequent section, all results from questionnaires, activity sensors, and interviews, are consolidated into an integrative synthesis, highlighting the new emergent themes that surface when looking across the various data sources. Participants reported initially experiencing a degree of discomfort when it came to engaging in unfamiliar dance activities with new people. However, the qualitative results indicate that this sense of uncertainty was quickly replaced by an increasing sense of security and feelings of acceptance. Participants adapted quickly to the new situations and felt progressively more comfortable in the social environment created by the dance activities. This supports the existing literature that links successful treatment and interventions with the sense of self-efficacy, that is formed by experiences of self-mastery (21, 54).

#### 4.3.1 Safety and acceptance

Participants initially reported some degree of discomfort in participating in unfamiliar dance activities with new partners. However, this sense of uncertainty was quickly replaced by a growing sense of security and acceptance. They quickly adapted to the new situations and felt more and more secure in the social environment that the dance activities created. The dance teachers also observed this positive development, noting an increased sense of safety and acceptance among the participants as they engaged more in the activities. The quantitative effect measurements showed an improvement in mental well-being, which seems to agree with these descriptions. The dance instructors reported observing positive development in the participants and noted an increased sense of security among them as they became more engaged in the dance activities. The comfort level was witnessed in the participants’ ease with wearing physical sensors, agreeing to be interviewed, and to participate in the study. It was therefore necessary to adopt a flexible approach to participation in the study.

#### 4.3.2 Community and equality

A central part of the adapted folk dance was a sense of community and equality, which many participants experienced across age, professionals, and participants. Through the communal dance sessions, hierarchies were broken down and participants experienced a sense of belonging and being part of something bigger. Professionals and participants noted how the dance created a new way of being with each other, where everyone participated equally and where relationships were strengthened across previously experienced boundaries. These observations support the quantitative measures for mental well-being and the improvement for the at-risk adolescents. No significant reduction in loneliness was observed, but the pre-mean for this group was relatively low and not clinically significant to begin with and raises the question of whether the measurement of loneliness was relevant for this population. Another consideration is that the potential for change is smaller and requires more participants, or perhaps takes longer before the individual experiences a change.

#### 4.3.3 Movement, flow, and energy

The quantitative results of the folk-dance intervention showed a significant increase in the time participants spent at normal and fast walking speeds. This suggests an increase in the level of activity and an increased physical exertion among participants. At the same time, the average resting period and heart rate were reduced, indicating an improved energy level and greater physical resilience in the participants. Describing their experiences of facilitating the folk dance, the dance instructors and musicians recounted how they focused on flow and on keeping the energy level in the room up, as they adapted to the participants’ needs to not become too self-conscious, and to be more attuned to the environment and allowing themselves to be guided by the company, the instruction, and the music.

#### 4.3.4 Mastery and self-confidence

Although participants experienced some physical challenges and fatigue during the dance activities, they also reported an increase in self-confidence and a sense of mastery. This was supported by observations from the dance instructors who noted small but significant progress in the participants that may not always be captured by the quantitative measurements. These results also align with the quantitative measurements of mental well-being for participants. Furthermore, the participants self-reporting of feelings of mastery and self-confidence relate to the basic psychological needs theory (BPNT), articulated by Deci & Ryan (54). BPNT is sub theory of self-development theory that links the three basic psychological needs—the need for competence, autonomy, and relatedness—as central to motivation for a given activity. Each of these needs are strongly related to social environments, and the sense of belonging as essential to wellness. These basic needs facilitate intrinsic motivation, and it appears from the qualitative results that these needs were met by the adapted folk-dance activity.

Because the intervention was offered in schools during regular school hours, and schools are important (if not essential) arenas for health promoting activities, it was important to also include other stakeholders such as teachers and the facilitators leading the intervention. The intervention allowed for different levels of participation at the comfort level of the individuals. The drawback of this flexibility is the lack of a control group, but the benefit is that we were able to identify specific challenges and solutions for conducting a comprehensive research study with a population that is very difficult to reach and for whom there is not much research available.

### 4.4 Insights for future studies

Developing interventions for at-risk adolescents in schools might require involvement from diverse community stakeholders (teachers, administrators, facilitators) and might pose different challenges than conventional studies that happen in hospitals or community centers. It is therefore important to work from a needs-based perspective to better understand how to meaningfully involve both the young people as well as other community stakeholders to ensure the intervention is feasible, meaningful, and sustainable. Work by Diaz presents concrete strategies for fully engaging at risk adolescents in their own health care (59). While there is evidence that exercise can positively benefit mental health by reducing anxiety (18, 36), it is also true that working with at-risk youth who suffer from anxiety, depression, and other mental health problems requires carefully considered approaches to data collection and research design to ensure the evaluation techniques themselves do not trigger unwanted or increased anxiety by creating more tense situations.

Our pilot project was developed in close cooperation with a professional arts organization and school instructors. Our results confirm the research showing that this demographic is particularly challenging when it comes to conducting intervention research (45). Of the five challenges identified by Trivedi-Batemen and Martingano for working with at-risk youth, we experienced all of them in this pilot study: access to participants, recruitment, attendance, participation, and data collection/data quality (45). Learning from these challenges, we found that trust, continued contact and engagement, and the role of schools and teachers in facilitating the intervention to be essential for both ensuring access to the young people and also for ensuring good data collection and day-to-day running of the intervention, and overall coordination with the research team.

Responding to the global trends of insufficient physical activity among adolescents, researchers have called for innovative solutions and an urgent scaling up of interventions that are known to be effective at increasing physical activity levels in adolescents (2, 13, 43). Ideally, these solutions should address the unique challenges young people face as they transition into higher education, employment, marriage, or parenthood, which are compounded for at-risk and vulnerable youth. However, understanding what makes interventions effective and sustainable can be difficult to assess. Of the 63 participants that attended the dance classes, only 55% (*n* = 35) participated in the post-interview, only 26% (*n* = 16) completed pre/post surveys, and only 6% (*n* = 4) agreed to wear activity monitors.

The decision to use a mixed methods research design was deliberately meant to address the challenges of this particular population, and to pilot test a high-quality and realistic methodology for comprehensively evaluating the effects of the intervention. Our multi-dimensional approach considered the feasibility of acquiring bio-signals through device monitoring (IMUs), self-reporting questionnaire data, and qualitative interview data while addressing the practical aspects of implementing adapted folk-dance sessions in schools, and collected the experiences of all those involved in the study. We found it extremely important to important to use a variety of measures and triangulation to ensure collecting sufficient data and the experiences of as many participants as possible while making them feel safe. Device-based measurements of physical activities for adolescents is generally scarce. Furthermore, there are not global standard protocols for reporting device-based measurements, and in general there is a lack of comparability of results that make it difficult to monitor and evaluate physical activity.

We found obtaining device-based measures for this particular population to be difficult. This can be attributed to the general challenge of recruitment associated with at-risk youths (42, 52), but also due to factors that are perhaps not as well understood (body image, general discomfort, stigma, privacy concerns, etc.). We learned that trust is essential, not only for establishing good rapport between the research team and the participants, but also for facilitating the research. The teachers played a valuable role by modeling trust and willingness to participate. Several teachers volunteered to wear IMUs themselves, which contributed to the feeling that everyone was on equal footing. (The movement data from the teachers is not reported in this study). We scheduled follow-up visits with the schools after the completion of the study to communicate the results and share the research findings. These visits were welcomed by the participants, who were eager to see their own individual data and progress. We can recommend that informational sessions for recruiting (e.g., giving a live demo of the IMU sensor) as valuable to help motivate individuals to participate in the study. We also strongly recommend follow-up visits when possible, as motivational mechanisms and to increase stakeholder involvement in research practice.

Finally, in contrast to much of the intervention research on physical activities for adolescents, the dance activities were structured but purposely adapted. This means that the physical activities and music were adjusted on the fly to the specific needs of the group, and the dance instructors and musicians were trained in adaptive techniques for vulnerable populations. We found this to be an especially important aspect for addressing the various needs and challenges of at-risk youth. The weekly interventions were tailored to match the progression and other challenges, such as efforts to include students who were reluctant to dance for the entire 90 min session, but were still motivated to participate. For future studies, we recommend continuing to explore the effect of adapted folk dance on a wider range of population groups, including other vulnerable and at-risk groups as well as preventive measures aimed at all age groups. It will also be valuable to implement studies with an active control group to evaluate the effectiveness of adapted folk dance in relation to other forms of physical activity and social interaction.

### 4.5 Limitations

As this was a pilot study, no power analysis was conducted beforehand. Study participants showed reluctance and uncertainty about using body sensors, participating in interviews, and being part of the study. Therefore, we had to adapt our approach to data collection to be both ethical and flexible. Although this meant some missing data, this pilot study yielded important insights concerning the specific challenges and possible solutions for collecting data from this population. Based on the interview feedback from 35 participants, mean participation in the six sessions was 70%. The reasons for such varied levels of participation were directly related to the challenges of this population: absenteeism, one participant entered late in the process, some participants were sick or had other tasks such as internships and doctor visits. While our study did demonstrate improvement in mental health, and physical fitness, we did not secure enough data from a sufficient number of participants to carry out a well-powered statistical analysis. No control or active control was used, which is a limitation as we do not know whether the suggested change is due to the dance, the break, the music, or the entire combination. Nor do we know if the possible change is due to, for instance, regular change of season or any other outside factors. While the number of study participants is relatively high compared with many dance interventions studies (53, 55), there were far too few study participants to suggest any statistically significant results. Moreover, we did not assess resting heart rate in our study. Instead, we assessed the heart rate during the resting periods of the folk-dance activity. Given the study participants’ reluctance to wear body sensors, we only obtained physiological data from four participants, which is a limitation. Future studies assessing proper resting heart rate are necessary to appropriately demonstrate the effects of dancing interventions on this parameter. Treatment fidelity was negatively compromised because of the risks and challenges associated with this demographic (e.g., high rates of absenteeism) and also because we did not strictly monitor attendance. For all these reasons, we must therefore be cautious with regards to the generalizability of our results.

## 5 Conclusion

Based on our multi-dimensional data collection and analysis, the results from this pilot study suggest that adapted folk-dance can improve mental health and well-being, and may positively contribute to physical fitness for at-risk adolescents by offering enjoyable physical activities. The intervention also had positive effects on the participants’ experiences of community, belonging, and confidence. This suggests the potential of folk-dance to promote physical health and well-being for at-risk adolescents in schools. The challenges we faced with recruitment and data collection confirmed that this is a difficult group to study, but with effort and by meaningfully involving the school and other stakeholders, these challenges can be addressed. Future studies would do well to prioritise attention to adherence, both for attendance and also to level of involvement throughout the activity, which are limitations in our study. Our results contribute to evidence-based research on dance as a health-promoting activity to address sedentary behavior and the associated challenges with mental well-being among at-risk adolescents. With sedentary behavior and poor mental health on the rise, it is crucial to explore and implement innovative approaches in schools. Adapted folk-dance can be a powerful tool to strengthen physical and mental health, and contribute to strengthening feelings of community and confidence for at-risk adolescents.

## Data Availability

The raw data supporting the conclusions of this article will be made available by the authors, without undue reservation.

## Appendix

**Table:**

Participant	Age	Girl	Boy	Participation%	Pre questionnaires	Post questionnaires	Both pre/post	Bodysensor	Interviews
FGUM6	20	1			1	1	1		
FGUM3	19	1		100	1	1	1		1
FGUM9	18	1		100	1	1	1		1
FGUM10	20		1		1				
FGUM2	21		1	83	1	1	1		1
FGUM11	17		1		1				
FGUM12	16	1		100	1				1
FGUM5	22		1	100	1	1	1	1	1
FGUM4	18	1		67	1			1	1
FGUM13	22	1				1			
FGUM14	18	1				1			
FGUM15	18	1				1			
FGUM16	19		1			1			
FGUH1	18		1		1				
FGUH2	19		1		1	1	1		
FGUH3	19		1		1	1	1		
FGUH4	18	1			1	1	1		
FGUH5	18		1		1	1	1		
FGUH6	19		1		1	1	1		
FGUH7	26		1		1				
FGUH8	21		1		1				
FGUH9	19		1		1	1	1		
FGUH10	17		1		1	1	1		
FGUH11	18	1			1	1	1		
FGUA1	16	1			1				
FGUA2	18	1		100	1	1	1		1
FGUA3	17	1		100	1				1
FGUA4	19	1		100	1				1
FGUA5	21	1		100	1			1	1
FGUA6	17	1		100	1				1
FGUA7	17	1			1				
FGUA8	28	1			1				
FGUA9	17	1		100	1				1
FGUA10	18		1		1				
FGUA11	22		1		1				
FGUA12	19		1		1				
FGUA13	17	1			1				
FGUA14	19	1			1	1	1	1	
FGUA15	28	1		100	1			1	1
FGUA16	17		1			1	1		
FGUA18	19	1		100		1		1	1
FGUA19	17	1				1			
FGUH12	18	1				1			
FGUH13	16		1			1			1
FGUH14	19		1			1			
FGUH15	17		1	33		1			1
FGUM16		1		17					1
FGUM17			1	50					1
FGUM19		1		17					1
FGUM20		1		17					1
FGUM21			1	50					1
FGUM22			1	67					1
FGUM23		1		50					1
FGUM24		1		17					1
FGUM25		1		33					1
FGUM26		1		100					1
FGUM27		1		100					1
FGUA20		1		83					1
FGUA21			1	67					1
FGUA22			1	67					1
FGUA23			1	100					1
FGUA24			1	17					1
FGUA25		1		100					1
**SUM**		35	28		35	26	16	6	33
**Mean**	19			71					
**Percentage**		56	44						
